# No Evidence for Role of Cutavirus in Malignant Melanoma

**DOI:** 10.3201/eid2508.190096

**Published:** 2019-08

**Authors:** Ulrike Wieland, Steffi Silling, Martin Hufbauer, Cornelia Mauch, Paola Zigrino, Frank Oellig, Alexander Kreuter, Baki Akgül

**Affiliations:** University of Cologne, Cologne, Germany (U. Wieland, S. Silling, M. Hufbauer, C. Mauch, P. Zigrino, B. Akgül);; Institute of Pathology, Mülheim an der Ruhr, Germany (F. Oellig);; Helios St. Elisabeth Hospital Oberhausen, University Witten/Herdecke, Witten, Germany (A. Kreuter)

**Keywords:** viruses, parvovirus, cutavirus, melanoma, skin, cutaneous, Germany

## Abstract

Cutavirus was previously found in cutaneous melanoma. We detected cutavirus DNA in only 2/185 melanoma biopsies and in 0/52 melanoma metastases from patients in Germany. Viral DNA was localized in the upper epidermal layers. Swab specimens from healthy skin were cutavirus positive for 3.8% (9/237) of immunocompetent and 17.1% (35/205) of HIV-positive men.

Cutavirus, a novel human protoparvovirus with linear single-stranded DNA, has been detected in fecal samples from children with diarrhea and in cutaneous T-cell lymphomas (CTCL) (*1*,*2*). Recently, Mollerup et al. reported the identification of cutavirus in 1 of 10 cutaneous malignant melanomas using viral enrichment methods with high-throughput sequencing and real-time PCR (*3*). This discovery raised questions concerning tropism and pathogenicity of cutavirus in human skin. We performed a retrospective study to determine cutavirus DNA prevalence and viral load in a large collection of formalin-fixed paraffin-embedded tissue biopsy specimens of malignant melanomas and in forehead swabs of healthy skin of immunocompetent and HIV-positive persons in Germany.

We used 185 cutaneous malignant melanoma biopsy specimens from 179 patients and 52 melanoma metastases from 42 patients from Germany for analyses with cutavirus real-time PCR (Appendix). We detected cutavirus DNA only in 2 nodular malignant melanomas, located on the abdomen of a 64-year-old man (MM-A) and on the cheek of an 85-year-old woman (MM-B). Viral DNA loads in these biopsies were 0.3 (MM-A) and 2.8 (MM-B) cutavirus DNA copies per β-globin gene copy. None of the 52 analyzed metastases carried cutavirus DNA (Table). The cutavirus PCR results of the 2 melanomas could be confirmed by sequencing and by in situ hybridization. In both melanomas, the cutavirus DNA–specific signals could be detected only in the superficial layers and on the surface of the skin but not in the tumor cells (Appendix Figure).

**Table:** Cutavirus DNA detection and DNA load in cutaneous malignant melanomas, melanoma metastases, and forehead swabs of healthy nonlesional skin from persons in Germany*

Sample type	No. samples analyzed	No. cutavirus DNA–positive samples† (%; 95% CI)	Median cutavirus DNA load (IQR)‡
Malignant melanoma tumor biopsies§¶	185	2 (1.1; 0.3–3.9)	0.30; 2.82#
Malignant melanoma metastases§**	52	0 (0; 0–6.9)	NA
Skin swabs of HIV-positive men§	205	35 (**17.1**; 12.5–22.8)	0.33 (0.66–3.81)
Skin swabs of healthy male controls§	237	9 (**3.8**; 2.0–7.1)	2.31 (0.19–11.72)

*Bold type indicates statistical significance. IQR, interquartile range; NA, not applicable.  †All samples were analyzed with CUTA-UPL5 real-time PCR as described in the Appendix. The formalin-fixed paraffin-embedded (FFPE) biopsies (melanomas and metastases) were also analyzed with 2 different real-time PCRs targeting the cutavirus nonstructural 1 gene (Appendix). These PCRs did not detect further cutavirus DNA–positive biopsies. ‡Cutavirus DNA load was determined in all cutavirus DNA–positive samples and was defined as cutavirus DNA copies per β-globin gene copy. §Details of the biopsies and skin swab specimens are provided in the Appendix. ¶From 21 cutavirus DNA–negative malignant melanomas, fresh frozen tissue could be analyzed in addition to the FFPE tissue samples (CUTA-UPL5-PCR). Cutavirus DNA was not detected in any of the 21 fresh frozen tissue samples. The cellular input of the fresh frozen tissue samples ranged from 1,230 to 40,600 β-globin gene copies per 2 μL extracted DNA (median 8,330, mean 10,892), indicating a high cellular input. #Shown here are the viral DNA loads found in the 2 cutavirus DNA–positive nodular malignant melanomas, MM-A and MM-B. **For 6 of the melanoma metastases, the primary tumor was also analyzed and was cutavirus DNA negative. The 2 patients with cutavirus DNA–positive melanoma biopsies (MM-A and MM-B) did not have metastatic disease.

To analyze the prevalence of cutavirus on healthy nonlesional skin, we used 442 forehead swab specimens from 237 immunocompetent men and 205 HIV-positive men that were available from a previous study (*4*) (Appendix). We found cutavirus DNA significantly more frequently on the skin of HIV-positive men than on the skin of healthy controls (17.1% vs. 3.8%; p<0.001 by 2-sided χ^2^ test; Table). Among HIV-positive men, we found a trend for a higher cutavirus prevalence in patients with AIDS compared with those without AIDS (14/59 [23.7%; 95% CI 14.7–36.0] vs. 19/140 [13.6%; 95% CI 8.9–20.2]; p = 0.078 by 2-sided χ^2^ test). The range of viral DNA loads found in the 44 cutavirus-positive skin swabs was 0.004–268.75 (median 0.41; interquartile range [IQR] 0.0–3.57); there was no significant difference between HIV-negative and HIV-positive men (p = 0.389 by Mann-Whitney-U test; Table).

Mollerup et al. found cutavirus DNA in 1 of 10 melanomas from Denmark and suggested investigating the role of cutavirus in cutaneous cancer (*3*). We detected cutavirus DNA in only 2 of 185 melanoma biopsy specimens and in none of 52 metastases. In situ hybridization localized the viral DNA on the surface of the 2 cutavirus-positive melanomas and not within the malignant cells. Our data therefore argue against an oncogenic role of cutavirus in malignant melanoma.

Väisänen et al. found cutavirus DNA in 2.9% of 136 skin biopsy specimens from 123 organ transplant recipients and in none of 159 skin biopsy specimens of 98 healthy adults (*5*). In accordance with Väisänen et al., we also found cutavirus more frequently in immunosuppressed patients than in healthy (immunocompetent) adults. Their finding related to healthy adults is in contrast to our results; however, we analyzed not skin biopsy specimens but widespread skin swab specimens covering ≈10 cm^2^ of forehead skin (*4*). Our cutavirus DNA prevalence data on normal skin of immunocompetent adults (3.8%) are in line with cutavirus IgG seroprevalence rates reported for adults in Finland, Iran, and Kenya (4.2%–5.6%). Lower cutavirus IgG seroprevalence rates have been found in the United States (0%) and Iraq (1%) (*6*).

A pathogenic role of cutavirus has been investigated in further malignancies. Concerning CTCL, conflicting results have been reported. Phan et al. have found cutavirus DNA in 23.5% (4/17) (*1*) and Väisänen et al. in 16% (4/25) of CTCL of the mycosis fungoides type (*5*). Our group recently analyzed 189 biopsies of various cutaneous B- and T-cell lymphoma types and detected cutavirus DNA only in 5.8% of 104 mycosis fungoides biopsy specimens (*7*). In contrast, Bergallo et al. could not detect cutavirus in 55 CTCL samples (*8*). The in situ hybridization results of a cutavirus-positive mycosis fungoides sample analyzed by Phan et al. pointed to a localization of the viral DNA in the superficial parts of the lesion (*1*), similar to the results we show. Therefore, it remains unclear whether cutavirus plays a role in the development of CTCL. Recently, Dickinson et al. could not detect cutavirus in oropharyngeal and oral cavity squamous cell carcinomas (*9*).

In summary, our data on cutavirus DNA prevalence and localization argue against an oncogenic role of cutavirus in malignant melanoma. However, oncolytic properties of this virus or viral hit-and-run oncogenesis cannot be excluded (*10*). Cutavirus seems to be more frequent on healthy skin of immunosuppressed patients than on the skin of immunocompetent persons and could be part of the human skin virome. It is possible that cutavirus is an apathogenic virus shed from human skin.

Appendix Materials and methods used in study of cutavirus in malignant melanoma.
