# Supplementary material for: No Evidence for Role of Cutavirus in Malignant Melanoma
Source: Emerg Infect Dis. 2019 Aug;25(8):1600–2. doi: 10.3201/eid2508.190096 (PMC6649328; doi:10.3201/eid2508.190096)
Supplement: Appendix — Materials and methods used in study of cutavirus in malignant melanoma. [file 19-0096-Techapp-s1.pdf]

Article DOI: https://doi.org/10.3201/eid2508.190096

No Evidence for Role of Cutavirus in Malignant Melanoma

Appendix

Materials and Ethics Statement

Formalin-fixed paraffin-embedded (FFPE) biopsies of 185 cutaneous malignant melanomas and 52 melanoma metastases were available for analyses by cutavirus real-time PCR. The 185 melanoma biopsies (69 superficial spreading, 45 nodular, 16 lentigo-maligna, 5 acral, and 50 malignant melanomas without further subtyping) were collected from 179 patients from Germany (70 females, 109 males) during 2002–2017. The patients´ mean age was 64.8 years (range 21–93). Because using fixed tissue can reduce the sensitivity of the PCR, matched fresh frozen tissue biopsies from 21 melanomas were analyzed in addition to the FFPE tissue samples. The 52 biopsies of melanoma metastases (28 subcutaneous, 16 lymph nodes, and 8 organ/tissue metastases) were collected from 42 patients (15 females, 27 males) during 2007–2017. The patients’ mean age was 68.2 years (range 39–96).

From a previous study, 442 forehead swabs from 237 healthy men and 205 HIV-positive men were available. Characteristics of the HIV-positive patients and of the control subjects have been described in detail (*1*). All men were Caucasians from Germany and were free of skin cancer, skin infections, and acute or chronic inflammatory dermatoses.

The study was approved by the ethics review boards of the University of Witten/Herdecke (no. 166/2017) and of the University of Cologne (no. 14–132).

Methods

Real-Time PCR

Cutavirus real-time PCRs were designed using the Roche Universal ProbeLibrary—Assay Design Center tool (https://lifescience.roche.com/en_de/brands/universal-probe-library.html-assay-design-center).

All samples (biopsies and skin swabs) were screened for cutavirus VP1 gene sequences using primers CUTA-UPL5 fw: 5′-AACCAAACACACCGAACCAG-3′ and CUTA-UPL5 rev: 5′-TGAAAAGGCTTACCTCTTTTGG-3′, together with locked nucleic acid (LNA) probe UPL#5: 5′-CAGCCACA-3′ (cat. no. 04685024001, Roche Diagnostics, https://www.roche.com).

In addition to CUTA-UPL5 PCR, all biopsies were analyzed with 2 further real-time PCRs targeting cutavirus NS1 gene sequences, CUTA-UPL48 PCR and a PCR using primers and probe as described by Mollerup et al. (*2*). CUTA-UPL48 PCR was performed with primers CUTA-UPL48 fw: 5′-CAAATTGAACCAAATGAAGTTGA-3′ and CUTA-UPL48 rev: 5′-CATGGATGTGTAGGCCTGTG-3′ with LNA probe UPL#48: 5′-ACTGGGAA-3′ (Roche, cat. no. 04688082001). β-globin gene PCR was performed from all samples to rule out DNA degradation caused by tissue fixation and to determine the cellular input. Primers β-403f: 5′-TGGGTTTCTGATAGGCACTGACT-3′ and β-532r: 5′-AACAGCATCAGGAGTGGAC AGAT-3′ with probe β-471pr: 5′-6FAM-TCTACCCTTGGACCCAGAGGTTCTTTGAGT-BBQ-3′ were used, as previously described (*3*), with one modification to the probe: the TAMRA quencher was replaced by a BlackBerry quencher (BBQ).

All PCR reactions (20 μL) were set up in Light Cycler 480 Probes Master (Roche) with 2 µl of extracted template DNA (QIAamp DNA Mini Kit, QIAGEN, https://www.qiagen.com). The UPL assays contained 0.2 μM of the respective forward and reverse primers and 0.1 μM of the respective Universal LNA Probe (UPL; Roche). For β-globin gene PCR, 0.3 μM of β-403f and β-532r and 0.2 μM β-471pr were used. Cycling conditions on a Light Cycler 480 II (Roche) were 95°C, 60 sec followed by 45 cycles 95°C, 10 sec; 60°C, 30 sec; 72°C, 5 sec. The real-time PCR according to Mollerup et al. (*2*) was set up in LightCycler 480 Probes Master reagent (Roche), including 500 nM target specific primers and 200 nM fluorescently labeled probes, 1–2.5 μL template DNA and H_2O ad 25 μL (*2*).

Negative control samples (human placental DNA) were included in each run and never yielded fluorescence signals above background. Ten-fold dilution series from 10^5 to 10 copies of cutavirus double-stranded (ds) DNA fragments (gBlock, IDT, https://www.idtdna.com) were used as standards and for calculation of the viral DNA copy number in cutavirus-positive samples. The slopes of the generated standard curves of the 3 cutavirus real-time PCR assays

demonstrated amplification efficiencies from 1.912 to 2.012. Cutavirus DNA load was defined as the number of cutavirus DNA copies per β-globin gene copy.

The analytical sensitivity of all real-time PCRs (CUTA-UPL5, CUTA-UPL48, Mollerup-PCR) was 1 DNA copy per reaction. Concerning the specificity of the CUTA-UPL5 assay, the forward primer does bind to several known cutavirus strains, as well as to the closely related bufavirus strains 1 and 3. The reverse primer contains 2 mismatches regarding bufavirus sequences. The CUTA-UPL48 forward primer and the LNA UPL48 contain mismatches concerning bufavirus 1 and 3 sequences, and the primers used in the real-time PCR described by Mollerup et al. (*2*) do not align with bufavirus sequences. We cannot exclude cross-reactivity of the CUTA-UPL5 and CUTA-UPL48 PCRs with bufavirus, but the specificity of the positive cutavirus PCR results of the 2 melanomas in our study was confirmed by sequencing and by in situ hybridization (Appendix Figure). Cutavirus-specific DNA sequences were found in skin swabs, as discussed later. Furthermore, bufavirus has so far been found only in fecal samples and in a nasal swab (*5*,*6*).

Confirmation of Positive Cutavirus PCR Results by Sequencing

The positive cutavirus PCR results of the 2 melanomas could be confirmed by sequencing (GenBank accession no. MK393127 for MM-B; 849 bp VP1/VP2 genes; 97% [825/849 bp] identity with cutavirus strain FR-D). Additionally, using VP1 primers described by Mollerup et al. (*2*), we obtained partial cutavirus VP1 gene sequences (306–522 nt) of different cutavirus strains (once each closely related to strains BR-337, BO-46, and FR-D, twice to strain CGG5–268) found in 5 forehead swabs of normal skin to confirm the specificity of the PCR results (GenBank accession nos. MK880125–MK880129).

References

1. Wieland U, Silling S, Scola N, Potthoff A, Gambichler T, Brockmeyer NH, et al. Merkel cell polyomavirus infection in HIV-positive men. Arch Dermatol. 2011;147:401–6. http://dx.doi.org/10.1001/archdermatol.2011.42

2. Mollerup S, Fridholm H, Vinner L, Kjartansdóttir KR, Friis-Nielsen J, Asplund M, et al. Cutavirus in cutaneous malignant melanoma. Emerg Infect Dis. 2017;23:363–5. http://dx.doi.org/10.3201/eid2302.161564

3. van Duin M, Snijders PJ, Schrijnemakers HF, Voorhorst FJ, Rozendaal L, Nobbenhuis MA, et al. Human papillomavirus 16 load in normal and abnormal cervical scrapes: an indicator of CIN II/III and viral clearance. Int J Cancer. 2002;98:590–5. http://dx.doi.org/10.1002/ijc.10232

4. Kreuter A, Hufbauer M, Silling S, Oellig F, Akgül B, Wieland U. Simultaneous induction of benign condyloma and high-grade anal dysplasia induced by low-risk human papillomavirus type 42. Acta Derm Venereol. 2018;98:616–7. http://dx.doi.org/10.2340/00015555-2913

5. Väisänen E, Fu Y, Hedman K, Söderlund-Venermo M. Human protoparvoviruses. Viruses. 2017;9:E354. http://dx.doi.org/10.3390/v9110354

6. Väisänen E, Mohanraj U, Kinnunen PM, Jokelainen P, Al-Hello H, Barakat AM, et al. Global distribution of human protoparvoviruses. Emerg Infect Dis. 2018;24:1292–9. http://dx.doi.org/10.3201/eid2407.172128

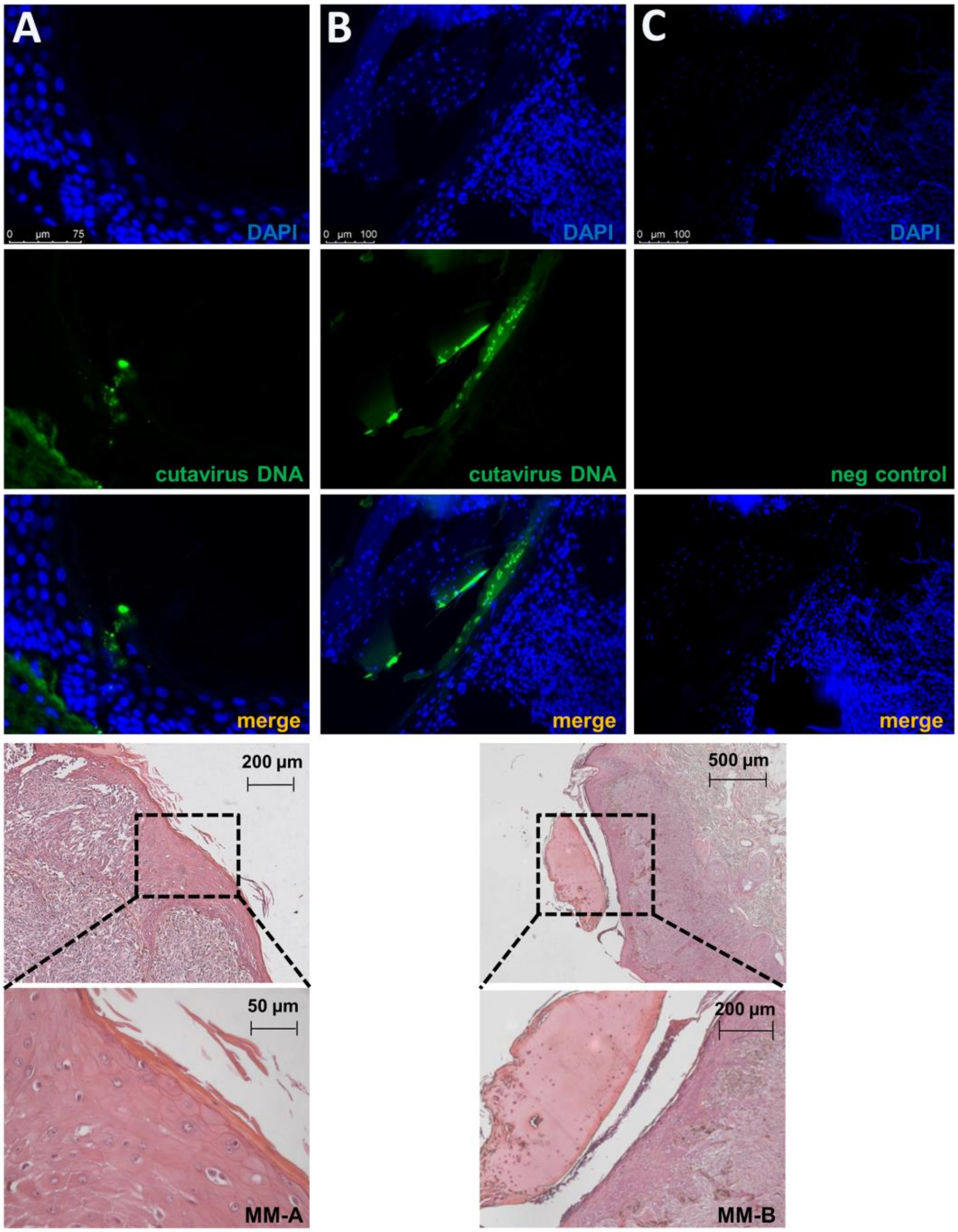

Appendix Figure. Detection of cutavirus DNA by in situ hybridization on the surface of two cutavirus PCR-positive malignant melanomas. A) Tumor MM-A; B, C) tumor MM-B. From top to bottom, a nuclear DAPI staining (blue), cutavirus DNA in situ hybridization (green, panels A and B; negative control in panel C), a merge of the green and blue fluorescent signals, and 2 different magnifications of routine histopathology (hematoxylin and eosin staining) highlighting the magnified tissue areas analyzed are shown. For the generation of the cutavirus probe, a dsDNA fragment (gBlock, IDT) spanning nucleotides 2505–2960 of cutavirus strain FR-D was cloned into the vector pJET1.2/blunt (Thermo Scientific,

https://www.thermofisher.com). The resulting cutavirus-specific vector and the empty vector (used as a negative control) were used as templates in DIG-Nick labeling reactions using the DIG-Nick translation mix (Roche Diagnostics). In situ hybridization of 4-μm sections was performed, as described previously (*4*). Detection of digoxigenin-labeled probes was achieved with anti-digoxigenin-POD Fab fragments (Roche) and the TSA Plus Fluorescin System (Perkin-Elmer, https://www.perkinelmer.com). In addition, sections were stained with 4′,6-diamidino-2-phenylindole (DAPI) (Merck, https://www.merck.com) and visualized by immunofluorescence microscopy.
